# Dynamics and function of the GET system microbial community: insights into the role of the genus *Bacillus* in biogas production

**DOI:** 10.3389/fmicb.2026.1729398

**Published:** 2026-02-09

**Authors:** Shaohua Chen, Hirotatsu Murano, Mio Matsushita, Sadanari Jindo, Tatsuya Hirano, Hiroto Tamura

**Affiliations:** 1 Graduate School of Agriculture, Meijo University, Nagoya, Aichi, Japan; 2 Faculty of Science and Technology, Meijo University, Nagoya, Aichi, Japan

**Keywords:** anaerobic fermentation, *Bacillus*, GET system, methane, rice straw, volatile fatty acids

## Abstract

**Introduction:**

To contribute to a sustainable society, we have established a novel technology for self-sufficient, renewable energy production called the GET system. This system produces approximately 300 liters of biogas from 1 kilogram of untreated rice straw in rice paddy fields while simultaneously reducing methane emissions from those paddies. A key feature of the GET system is that 2nd addition of rice straw, made 2 months later, significantly increased biogas production compared to the initial addition. However, no volatile fatty acids (VFAs)—key substrates for methanogens—were detected after 2nd addition of rice straw.

**Methods:**

To understand this phenomenon, the microbial community in the GET system at various time points was analyzed using next-generation sequencing (NGS) and real-time quantitative PCR (RT-PCR).

**Results:**

Volatile fatty acids (VFAs), particularly acetic acid, are important substrates and indicators for methane production. However, in this study, VFAs, including acetate, were not detected after 2nd addition of rice straw, which significantly increased biogas production. In the analysis of microbial community structure, although bacteria from Clostridium and methanogenic archaea are often considered to play a dominant role in anaerobic cellulolytic fermentation and methanogenesis, respectively, the GET system was dominated by Bacillus, which had an average abundance of 23.8%. This abundance increased fourfold after 2nd addition of rice straw, mainly due to the increased presence of *B. fumarioli* under strict anaerobic condition, which has been recently transferred into the genus *Neobacillus* (Patel and Gupta, 2020). However, the average abundance of *Methanosaeta* and *Clostridium* accounted for 3.7 and 7.1% of the total, respectively, with no significant changes in abundance throughout the experimental period.

**Discussion:**

The synchronization of the increase in *B. fumarioli* abundance with the increase in biogas production in the GET system indicated that *B. fumarioli* plays a key role in maintaining a perfect balance with the methanogenic archaea *Methanosaeta* by decomposing rice straw, subsequently producing VFA, and ultimately generating acetate, which serves as a substrate for methane production.

This study provides the first functional insight into the role of *B. fumarioli* in efficient methane production under strictly anaerobic conditions.

## Introduction

Methane emissions, particularly those originating from paddy fields, have been identified as a significant source of greenhouse gases contributing to global warming (Jackson et al., 2020). The prevalent anaerobic conditions in these paddy fields create an optimal environment for methane production, with the straw residue serving as the primary substrate for methane-producing microorganisms (Mussoline et al., 2013). To mitigate these emissions, researchers have explored various strategies, such as alternate wetting and drying (AWD) and mid-season drainage (MSD) (Ishfaq et al., 2020). Diverging from the traditional approach of how to control methane emissions from paddy fields, we have leveraged the principles and characteristics of methane production in paddy fields to develop a technology named The GET system. The GET system is an efficient production system of biogas (G) with a methane concentration of 60% as renewable energy (E) from a *tanbo* (T), which means paddy field in Japanese (Chen et al., 2020). Since methane is a clean energy source and the main component of natural gas, the GET system is a new renewable energy technology that produces biogas on-site with high efficiency, utilizing rice paddies as a natural fermentation tank and rice straw as a fermentation substrate, thereby eliminating the need for conventional fermentation plants. Moreover, the resulting 25.7% of carbon in rice straw was simultaneously stored in the soil as fermentation residue, improving soil fertility and creating a carbon sink. The GET system is therefore a game-changing technology, offering a potential solution to reduce atmospheric greenhouse gases (GHG) (Chen et al., 2020).

Therefore, the GET system simultaneously addresses three critical issues in advancing low-carbon sustainable development globally: methane emissions from paddy fields, rice straw waste management, and the generation of renewable energy. Our previous study confirmed that the GET system could produce up to 300 L biogas kg^–1^ untreated rice straw, with a methane concentration of more than 60% at the optimum amount of 14 kg rice straw m^–2^, under operating parameters, pH 6.0–6.6, redox potential below −200 mV, and fermentation temperature between 20 and 30°C (Chen et al., 2020). However, the mechanism by which the GET system can efficiently produce biogas under such an environment remains unclear.

Although recent research has begun to extensively study the decomposition process and community structure of methanogens (Guo et al., 2018; Liu et al., 2023; Wang X. et al., 2022), the complex process of rice straw decomposition, particularly that of cellulose, remains contentious. Therefore, elucidating the microbial community structure involved in rice straw decomposition and methane fermentation in GET systems will lead to improving and expanding the renewable energy production capacity of GET systems.

In traditional anaerobic digestion processes (Magdalena et al., 2019), the concentration of volatile fatty acids (VFAs) has been widely acknowledged as a crucial parameter for assessing the anaerobic biodegradability of substrates. Notably, acetic acid is a pivotal substrate for methanogenesis, exerting a significant influence on the proliferation and metabolic functions of the methanogenic microbial consortium. Since the GET system achieves highly efficient biogas production of 300 L/kg of rice straw simply by mixing unprocessed rice straw with soil every 2 months (Chen et al., 2020), this study aimed to clarify the reasons for this high productivity through quantitative analysis of VFAs and analysis of microbial community structure using next-generation sequencing (NGS) technology and real-time quantitative PCR.

## Materials and methods

### Concept of microbial community structure analysis

**FIGURE 1:**
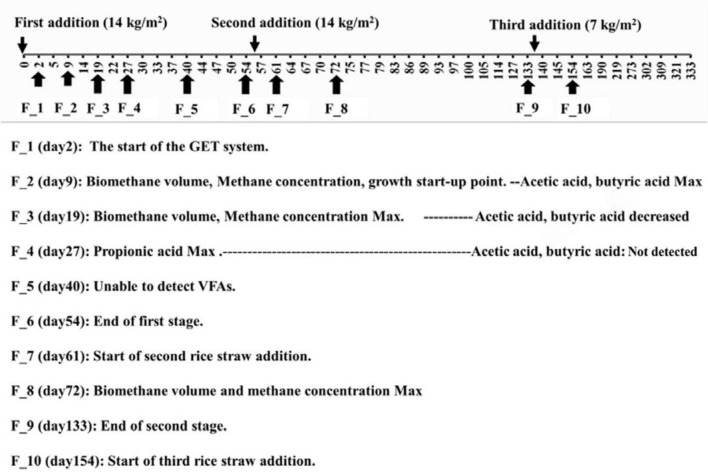
Experimental design and rationale for soil sampling points.

In the GET system, biogas production and methane concentration increased markedly immediately after the 2nd addition of rice straw,as our previous research (Chen et al., 2020). To resolve the microbial and metabolic processes underlying this characteristic response, soil samples were collected at 3–5-day intervals. This sampling strategy was designed to capture short-term fluctuations in volatile fatty acids (VFAs) and corresponding shifts in microbial community structure during the early stages of substrate turnover (Figure 1).

The next-generation sequencing (NGS) based on DNA extracted from soil samples was used to observe the microbial community structure involved in rice straw decomposition and methane fermentation in GET systems, which provides a comprehensive overview of taxonomic structure across sampling points. However, because DNA-based NGS profiles include both active and inactive populations, additional approaches were required to evaluate metabolically active microorganisms and to validate temporal changes suggested by high-throughput sequencing.

Therefore, denaturing gradient gel electrophoresis (DGGE) was employed as a complementary method to visualize dominant and dynamically changing microbial populations across sampling points. DGGE enables direct comparison of banding patterns over time and serves as an effective tool for confirming major shifts in community structure, particularly for targeted microbial groups, prior to quantitative analysis.

To further assess microbial activity, total RNA was extracted from the same soil samples and reverse-transcribed into cDNA. Real-time quantitative PCR (qPCR) using specific primers was then performed to quantify temporal changes in RNA-derived gene copy numbers of selected taxa and functional groups. RNA-based qPCR allowed transcriptionally active populations to be distinguished from dormant or residual DNA, enabling interpretation of gene copy number dynamics as indicators of microbial activity rather than mere presence.

By integrating VFA profiles with NGS-based community structure, DGGE-based pattern validation, and RNA-based qPCR activity data, this study established a multi-layered analytical framework to evaluate the reason why the efficiency of the GET system differed substantially following the 2nd rice straw addition (Supplementary Table S2).

### Set GET system

The construction method of GET system was mentioned in our previous paper (Chen et al., 2020). In briefly (Figure 1), cut the sun-dried rice straw into approximately 15 cm lengths and mixed it with the paddy soil on the paddy field, then constructed a gas sampling bed (4.5 m in length, 0.8 m in width, and 0.2 m in height) consisting of a gas production bed (L: 3.0 m × W: 0.8 m × H: 0.2 m) and a soil sampling bed (L: 1.0 m × W: 0.8 m × H: 0.2 m) using rotary tiller. The pH and oxidation-reduction potential (Eh) meters (Orion 3-Star plus, Thermo Fisher Scientific, Waltham, MA, United States) and the soil pore water collector, Mizutor (DIK-8392, Daiki Rika Kogyo, Saitama, Japan) were installed at the same height as the ground in the center of the gas sampling beds. The gas and soil sampling beds (i.e., fermentation beds) were covered with an impermeable rubber sheet (10 m in length × 2.0 m in width × 1 mm in thickness) (Taiyo Kogyo, Osaka, Japan), and sealed the edges with soil. After connecting the gas collection apparatus through the interface on the top of the rubber sheet, the experimental area was flooded to make anaerobic fermentation beds and to seal the gas sampling beds using water pressure. The experiments were performed with three beds.

### Experimental design and sampling strategies

Three additions of rice straw at intervals of 2 months were performed as follows: the 1st addition was 14 kg/m^2^, the 2nd addition was 14 kg/m^2^, and the third addition was 7 kg/m^2^ (Figure 1). Samples for analysis of biogas volume, methane concentration in biogas, and concentration of VFAs were taken at intervals of 3–5 days after rice straw addition. Biogas, soil, and VFAs samples were collected from each of three fermentation beds operated in parallel.

Soil samples from F_1 to F_10 (Figure 1) were collected based on the dynamics of biogas volume, and concentrations of VFAs and methane.

### Volatile fatty acids analysis

Soil pore water samples (2 mL) were collected from the gas sampling beds using a soil pore water collector. To purify the water samples, an equal volume of chloroform was added to the water samples, followed by vortex mixing (20 s × 3 with 5 s intervals) and centrifugation (3,000 rpm, 15 min, 25 °C). The aqueous supernatant was recovered and filtered through a 0.2 μm PTFE membrane filter (DISMIC-13CP, Advantec Toyo Kaisha, Tokyo, Japan).

Volatile fatty acids were quantified using a high-performance liquid chromatography (HPLC) organic acid analysis system equipped with an electrical conductivity detector (Prominence, Shimadzu, Kyoto, Japan). Separation was performed on two serially connected Shim-pack SCR-102H columns (300 mm × 8.0 mm i.d.) with a Shim-pack SCR-102H(G) guard column (50 mm × 7.8 mm i.d.) maintained at 40 °C. The injection volume was 50 μL and the autosampler was set to 4 °C. The mobile phase consisted of (A) 5 mM sodium *p*-toluenesulfonate and (B) 5 mM sodium *p*-toluenesulfonate containing 20 mM Bis-Tris and 100 μM EDTA, delivered at 0.8 mL/min for both pumps (A and B). The total run (analysis) time was 55 min.

Quantification was performed by the external standard method using mixed organic acid standards (pyruvic acid, glyoxylic acid, lactic acid, acetic acid, propionic acid, butyric acid, isovaleric acid, succinic acid, and formic acid), and concentrations were reported as mg/L. Detailed instrument settings are provided in Supplementary Table S1.

### DNA and RNA extraction

DNA and RNA were co-extracted from soil using the FastDNA™ SPIN Kit for Soil (MP Biomedicals; catalog no. 6560200) following the manufacturer’s protocol, with minor modifications as described by Tournier et al. (2015). Briefly, 250 mg of soil (instead of 500 mg recommended by the kit) was placed in a Lysing Matrix E tube and pre-frozen at −80°C overnight. Cells were lysed by bead beating (two cycles, 40 s each at 6.0, with cooling on ice between cycles), and the lysate was clarified by centrifugation. DNA was purified from the Binding Matrix fraction according to the kit instructions and eluted in 150 μL of DES. The corresponding supernatant was used for RNA purification following the kit-based procedure (including alcohol precipitation and RNase-free handling), and RNA was finally resuspended in RNase-free water.

### cDNA synthesis

The cDNA was synthesized using the High-Capacity cDNA Reverse Transcription Kit (Applied Biosystems, Thermo Fisher Scientific, Waltham, MA, United States) according to the manufacturer’s protocol.

### PCR-DGGE

PCR amplification for DGGE targeting *Clostridium* was performed in 50 μL reactions using KOD One^®^ PCR Master Mix (TOYOBO, Osaka, Japan) with primers Chis150f (5′-AAAGGRAGATTAATACCGCATAA-3′) carrying a 5′ GC-clamp and ClostIr (5′-TTCTTCCTAATCTCTACGCA-3′). Thermal cycling conditions were: 98 °C for 5 min; 35 cycles of 98 °C for 5 s, 62 °C for 5 s, and 72 °C for 10 s; followed by 72 °C for 3 min. Amplicon size and specificity were confirmed by electrophoresis of 5 μL PCR product on 2% (w/v) agarose gels.

Denaturing gradient gel electrophoresis (DGGE) was carried out using a DCode Universal Mutation Detection System (Bio-Rad Laboratories, Hercules, CA, USA). PCR products (50 ng per lane) were separated on 10% (w/v) polyacrylamide gels containing a 10–60% denaturing gradient for *Clostridium*. Then, 100% denaturant was defined as 7 M urea and 40% (v/v) formamide. Gradients were generated using a Model 475 Gradient Delivery System (Bio-Rad). Electrophoresis was performed in 1 × TAE buffer at 58 °C with a pre-run at 160 V for 10 min, followed by electrophoresis at 160 V for 12 h for *Clostridium* DGGE profiles.

After electrophoresis, gels were stained with SYBR Gold (Thermo Fisher Scientific) for 30 min with gentle agitation and visualized under UV illumination. Dominant bands of interest were excised, and DNA was eluted overnight in 50 μL sterile water. The recovered DNA was re-amplified in 20 μL reactions using KOD One^®^ PCR Master Mix with primers 150f (5′-AAAGGRAGATTAATACCGCATAA-3′) and ClostIr (5′-TTCTTCCTAATCTCTACGCA-3′) under the following cycling conditions: 98 °C for 5 min; 30 cycles of 98 °C for 5 s, 61.5 °C for 5 s, and 72 °C for 10 s; followed by 72 °C for 3 min.

PCR products were purified using the QIAquick PCR Purification Kit (QIAGEN, Hilden, Germany) according to the manufacturer’s instructions. Purified amplicons were outsourced for sequencing. Purified samples were sent to the analysis company for sequencing. The determined nucleotide sequence was searched for homology by NCBI BLAST.

### Next-generation sequencing

Extracted genomic DNA was used for Illumina MiSeq library preparation and paired-end sequencing. The V3–V4 region of the 16S rRNA gene was amplified using primers 341F (5′-CCTACGGGNGGCWGCAG-3′) and 805R (5′-GACTACHVGGGTATCTAATCC-3′) to characterize microbial community structure and diversity in the GET system.

Raw reads were quality-filtered using Trimmomatic v0.38 with a sliding-window approach; bases were trimmed when the mean Phred score within the window fell below Q20, and reads shorter than 50 bp after trimming were discarded. Adapter and primer sequences were removed using Cutadapt v1.16.

Denoising and amplicon sequence variant (ASV) inference were performed using DADA2 implemented in QIIME2 (QIIME2 v2020.8.0; DADA2 v2020.8.0). Quality filtering was conducted with maxN = 0 and expected error thresholds of maxEE = 2 (forward reads) and maxEE = 3 (reverse reads). Error models were learned from the data using the learnErrors function, followed by dereplication and ASV inference. Paired-end reads were merged with a minimum overlap of 12 bp, requiring identical sequences across the overlap region, and chimeric sequences were removed using the consensus method.

Taxonomic classification of representative ASV sequences was performed using mothur (v1.39.5; classify.seqs) against the Greengenes and RDP reference databases with a confidence threshold of 0.6. ASVs without taxonomic assignments were removed. In addition, ASVs assigned to non-target groups (e.g., archaeal assignments in bacterial 16S datasets) were excluded prior to downstream analyses.

### Sequencing

Purified samples were sent to the analysis company for sequencing. The determined nucleotide sequence was searched for homology by NCBI BLAST.

### Real-time PCR

Real-time PCR was performed by Step One Plus™ (Applied Biosystem, Thermo Fisher Scientific) to quantify gene transcription. The sequences of the target genes and the primers for amplifying them were shown in Supplementary Table S2. The reaction composition and reaction conditions of real-time PCR were shown in Supplementary Table S3. For the preparation of the calibration curve, the plasmid DNA prepared in the preparation of the standard fragment and the genomic DNA of the *C. butyricum* Prazmowski 1880 strain and the *B. cereus* strain CCM2010 were used. All experiments were performed in triplicates, and the results were expressed as mean ± standard deviation.

## Results

### Dynamics of VFAs in the GET system

Volatile fatty acid (VFA) concentrations are widely recognized as a key parameter for evaluating anaerobic digestion processes (Magdalena et al., 2019). Acetate, in particular, is a crucial substrate for methane production, influencing the proliferation and metabolic function of methanogenic microbial communities. Therefore, this study first performed quantitative analysis of VFAs and examined the overall structural evolution of the microbial community based on sequence data generated through the aforementioned experimental protocol.

**FIGURE 2:**
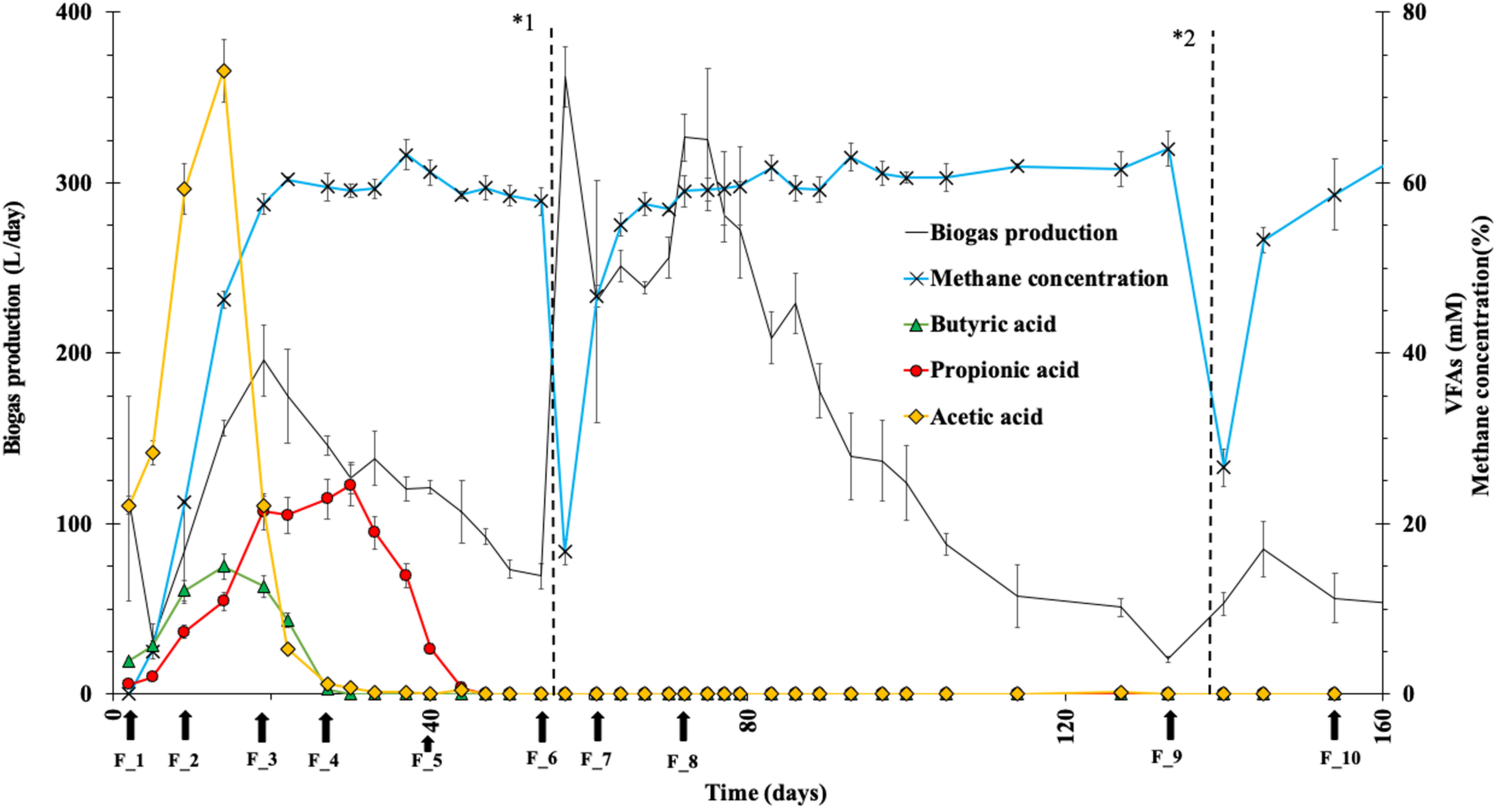
The quantitative analysis of VFAs, biogas production, and methane concentration in the GET system. Biogas production (black line, left y-axis); methane concentration (blue line with crosses, right y-axis); acetic acid (yellow diamonds, right y-axis); propionic acid (red circles, right y-axis); butyric acid (green triangles, right y-axis). Arrows indicate the soil sampling time point (F_1–F_10). Vertical dashed lines (1 and 2) indicate the second and third rice straw addition time points. (Values were presented as mean ± SD, *n = 3*). *1: Time for the second rice straw addition. *2: Time for the third rice straw addition.

In the GET system, three VFAs were mainly detected (Figure 2), i.e., acetic acid, propionic acid, and butyric acid. Within 5 days of the start of the experiment concentrations of acetic acid and butyric acid increased sharply and reached the maximum of 75 and 15 mM, respectively, on day 9 (F_2). Their concentrations then rapidly decreased, reaching nearly zero by day 22 and becoming undetectable on day 40 (F_5). Conversely, propionic acid displayed a more gradual increase, reaching its highest concentration (20 mM) on day 27 (F_4). Although propionic acid persisted for the longest period among the four acids detected, propionic acid also mirrored the decline from day 30, plummeting to zero on day 40 (F_5).

Following the additional batch of rice straw, no VFAs were detected although the biogas production rate was significantly increased, and the concentration of methane reached 60% on day 19 (F_3) and remained constant thereafter despite no detectable VFAs (Figure 2). This indicated that the rice straw was quickly hydrolyzed to monosaccharides such as glucose, and the resultant monosaccharides were converted to VFAs, which were then rapidly utilized as substrates for methane production without their accumulation.

Since the VFAs are essential intermediates for methanogenesis in the anaerobic fermentation process, therefore, our results encourage elucidation of the microbial community structure in the GET system.

### The classifications based on NGS results

#### Phylum-level classification and clustering

**FIGURE 3:**
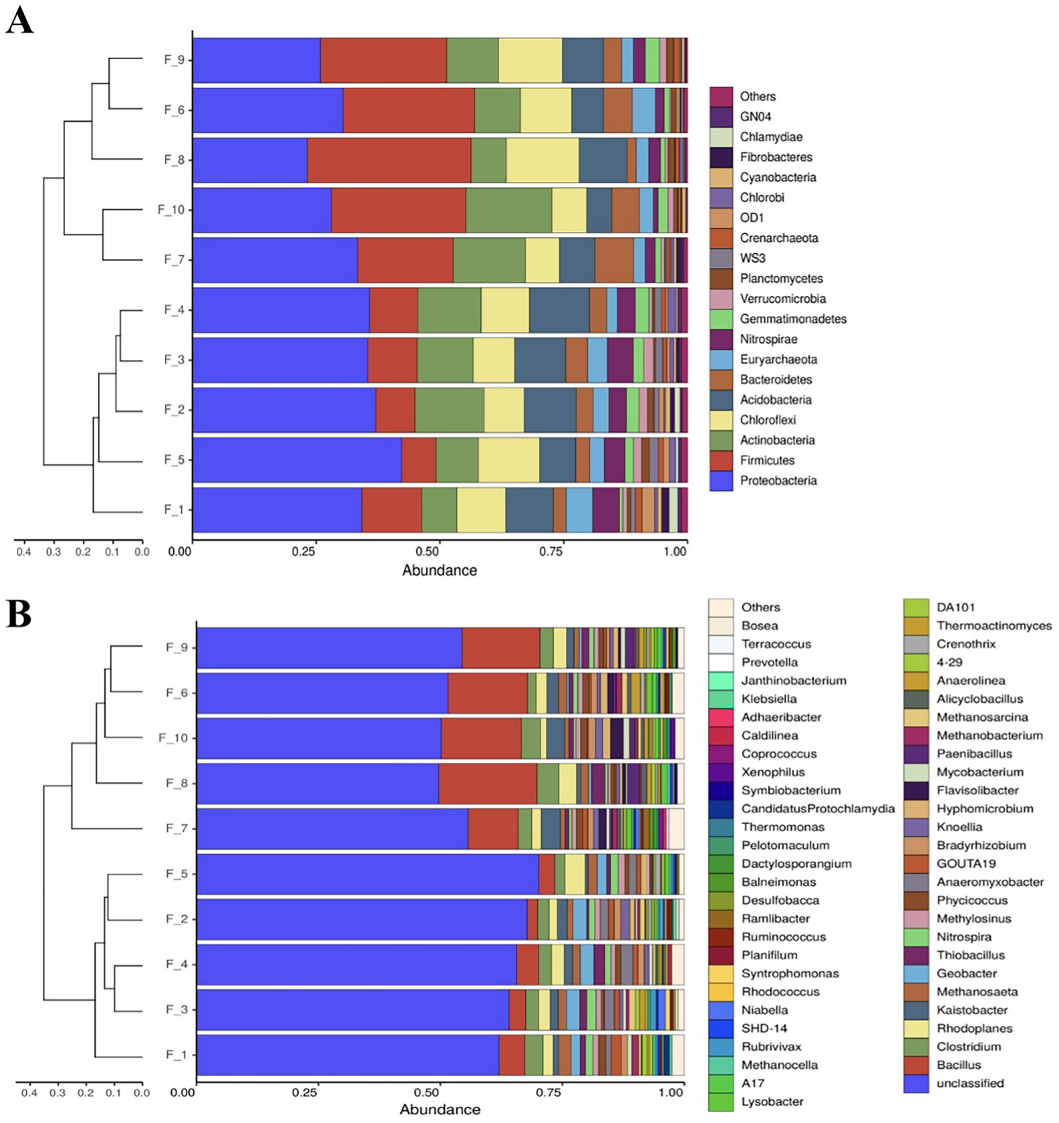
Clustering trees and histogram combination analysis charts of phylum-level **(A)** and genus-level **(B)**.

At the phylum level, 34 phyla were identified, of which the following five phyla were predominant: Proteobacteria (32.5%), Firmicutes (17.8), Actinobacteria (11.2%), Chloroflexi (10%), and Acidobacteria (8.6) being the dominant groups, accounting for over 9% of the optimized sequence totals. Other less abundant phyla, such as Bacteroidetes, Euryarchaeota, and Nitrospirae, were also found in most samples (Figure 3A).

Although Proteobacteria was the dominant phylum with the largest proportion in the GET system, its abundance tended to decrease after the 2nd addition of rice straw. In contrast, the abundance of phylum Fimicutes increased significantly (Figure 3A).

Through the clustering relationship, the biggest difference between the two groups came from the four-fold increase in the abundance of the Firmicutes phylum after the 2nd addition of rice straw (Figure 3A). Although F_6 was the last stage of the fermentation process after the 1st addition of rice straw, due to the significant increase in the abundance of phylum Firmicutes in the F_6, the clustering relationship classified the F_6 into the group after the 2nd addition of rice straw, indicating that the enrichment of the suitable microbial community structure in the GET system had completed the change at the end of 1st fermentation stage (F_6).

#### Genus-level classification and clustering

At the genus level analysis of the GET system (Figure 3B), a total of 54 genera were identified across all samples. The largest proportion of abundance belonged to unclassified genera, which accounted for an average abundance of 59%, predominantly originating from the phylum Proteobacteria. After the 2nd addition, unclassified genera decreased as *Bacillus* increased.

The abundance of *Bacillus* increased from 3.2% at F_5 to 16.2% at F_6. Following the 2nd addition of rice straw, the average abundance of *Bacillus* remained around 15%, which was about five times higher than after the 1st addition of rice straw. In contrast, the abundance of *Clostridium* did not show significant variation throughout the experiment, consistently maintaining around 2%. The fact that *Bacillus* was the primary factor influencing the accumulated microbial community structure in the GET system suggests that it might be involved in the significant increase in biogas production after the 2nd addition of rice straw. In the GET system, acetoclastic *Methanosaeta* was the only dominant methanogen at the genus level, with a mean abundance of 1.5%. This abundance was higher after 1st addition of rice straw than after 2nd addition of rice straw (Figure 3B).

#### Species-level quantitative analysis based on real-time PCR

Real-time quantitative PCR (qPCR) is a crucial method for quantifying specific DNA or RNA sequences, playing a key role in analyzing microbial community composition changes. Since the copy number of ribosomal DNA varies slightly depending on the bacterial species, some error occurs during the PCR amplification stage. However, it is known that there are problems in determining the general composition ratio of the microbial consortium. Therefore, it enables precise detection and quantification of various microorganisms, offering insights into their population dynamics under different conditions. By utilizing specific genetic markers, quantitative PCR (qPCR) provides insight into the composition and fluctuations of microbial communities, thereby enhancing our understanding of their ecological and industrial roles. To confirm the results of the NGS analysis in this study, qPCR was performed for designated microbes of the genera *Bacillus*, *Clostridium*, and *Methanosaeta*.

#### Bacillus

By analyzing the species-level classification in NGS data (Supplementary Figure S2), two dominant classified species within *Bacillus* were identified as *B. fumarioli* which has been recently transferred into the genus *Neobacillus* (Patel and Gupta, 2020) and *B. flux*. Based on the sequences provided by the NGS results, specific primers were used to conduct absolute quantitative analysis on these two species (Supplementary Table S2).

**FIGURE 4:**
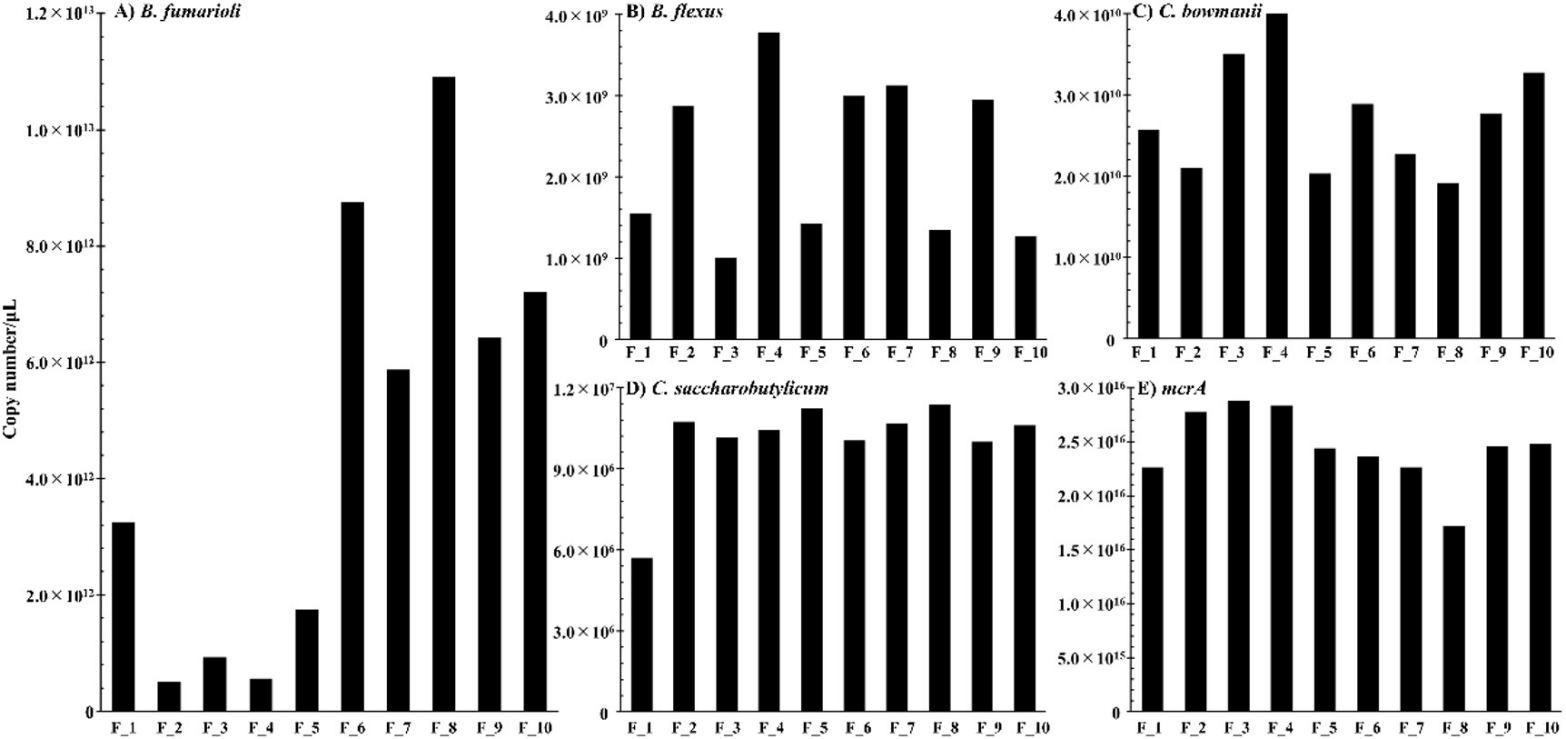
Species-level quantitative analyses of microbial community structure in the GET system. **(A)** *B. fumarioli* (This species has been recently transferred into the genus *Neobacillus*, Patel and Gupta, 2020); **(B)** *B. flexus*; **(C)** *C. bowmanii*; **(D)** *C. saccharobutylicum*; **(E)** *mcrA*.

As a result, the copy number of *B. fumarioli* remained at lower levels throughout the 1st fermentation stage (Figure 4A, F_1–F_5). However, at the end of the 1st addition of rice straw (F_6), the copy number of *B. fumarioli* had increased by 4 times compared to F_5. Following the 2nd addition of rice straw, it slightly decreased to 6 × 10^12^ and then surged significantly (Figure 4A). This change in *B. fumarioli*’s copy number aligned with the dynamics in *Bacillus* abundance and the biogas production in the GET system after the 2nd straw addition (Figure 2). The significant increase in biogas production, despite the absence of detectable VFAs after the 2nd addition of straw, was attributed to an evident increase in the population of *B. fumarioli* in the microbial community in the GET system. Thus, *B. fumarioli* was a key player affecting the rate and volume of biogas production within the GET system.

On the other hand, the copy number of *B. flexus* was 1,000-fold lower than that of *B. fumarioli* and fluctuated significantly throughout the fermentation process (Figure 4B). Although there is another increase at F_6, it did not surpass the maximum value of the first fermentation stage. Its fluctuation pattern did not correspond to the changes observed for *Bacillus* in the NGS results. Moreover, since this copy number fluctuation was not coupled to biogas production, *B. flexus* was not the primary factor influencing methane production in the GET system.

##### Clostridium

*Clostridium* were known to contribute to cellulose degradation and acid production under anaerobic environments. However, microbial community analysis using NGS (Figure 3B) showed that *Clostridium* were less effective in the GET system than *Bacillus*. To confirm this, specific primers targeting *Clostridium* were designed and the results were verified using DGGE and quantitative PCR.

Through the analysis of species-level classification in DGGE data (Supplementary Figure S1; Supplementary Table S4), *Clostridium* species with the highest and higher homology were identified as *C. saccharobutylicum* (99.38%) and *C. bowmani* (98.19%), respectively. Using specific primers (Supplementary Table S2), absolute quantitative analyses were conducted for these two species. Subsequent observations revealed that *Clostridium* had the lowest copy number among the three genera and the copy number of *C. bowmani* was 10^4^ higher than that of *C. saccharobutylicum* (Figures 4C,D). The copy number of *C. bowmani* showed an increasing tendency after the 1st addition of rice straw, but after 2nd addition of rice straw addition, when biogas production increased significantly, the copy number of *C. bowmani* did not increase but rather decreased (Figure 4C). Furthermore, it was not correlated with changes in VFAs.

The copy number of *C. saccharobutylicum* was steady at the lowest copy number among the tested microbes, ranging from 10^6^ to 10^7^ throughout the experiment, and did not correlate with the break of the biogas production right after the 2nd addition of rice straw (Figure 4D).

##### Archaea

The *mcrA* gene (methyl-coenzyme M reductase alpha subunit gene) encodes the alpha subunit of the enzyme methyl-coenzyme M reductase, which reduces methyl-coenzyme M to methane in the final step of the methane synthesis pathway in methanogenic archaea (Springer et al., 1995; Luton et al., 2002; Juottonen et al., 2006; Gagnon et al., 2011). Due to its central role in methane production, the *mcrA* gene is often used as a molecular marker to study methanogenic archaea in environmental samples (Luton et al., 2002). Therefore, the abundance of the *mcrA* gene detected by quantitative PCR can estimate the activity and diversity of methanogenic archaea (Morris et al., 2016). Interestingly, in the GET system, NGS data indicated that the only predominant genus classified was *Methanosaeta* (Figure 3B). Therefore, the quantitative PCR results likely reflected the change only in *Methanosaeta*, despite the primers were designed based on the *mcrA* gene (Supplementary Table S2).

The quantitative analysis of the *mcrA* gene revealed that among the treated microorganisms, the copy number of archaea had the highest copy number at the order of 10^16^ (Figure 4E). After the 1st addition of rice straw, the copy number of archaea slowly increased during the initiation phase of fermentation but began to decline after F_4. Following the 2nd addition of rice straw, this decline continued, reaching its lowest point at F_8, when biogas production and methane concentration conversely reached their highest values, after which it rebounded and remained stable at 2.5 × 10^16^. Throughout the fermentation process of the GET system, the range of variation in archaea was not significant and was less than two-fold. After the 2nd addition of rice straw, in particular, the methane concentration in the GET system increased substantially, but the abundance of archaea declined, indicating that archaea were not responsible for the rapid and substantial methane production following the 2nd addition of rice straw.

## Discussion

In the GET system, rice straw was incorporated into paddy soil at the experimentally validated optimal addition rate (14 kg/m^2^), enabling on-site biomethane production of approximately 300 L/kg of rice straw with a methane concentration of ∼60% (Chen et al., 2020). A notable operational feature of the GET system is the pronounced increase in biogas production following the 2nd rice straw addition (Figure 2; Chen et al., 2020). Accordingly, the present study focused on this “second-addition response” and examined the associated microbial and metabolic characteristics by analyzing VFAs and microbial community structure. Data obtained after the third addition were not discussed in detail because the decrease in temperature under field conditions reduced microbial activity and biogas production, making direct comparison with the characteristic second-addition response inappropriate.

VFAs concentrations increased rapidly during the early phase after the 1st addition of rice straw (F_2) (Figure 2), which was in agreement with accelerated hydrolysis and acidogenesis of lignocellulosic substrates under anaerobic conditions. Similar transient VFAs accumulation has been reported in anaerobic digestion systems, where rapid release of fermentable compounds from plant biomass leads to a temporary increase in VFAs (Khantibongse and Ratanatamskul, 2023). Previous studies have also shown that accumulation of VFAs, particularly acetate, can cause acidification and suppress methanogenic activity, ultimately reducing methane yield (Wang S. et al., 2022). In contrast, in the GET system, biogas production was not inhibited during the initial period of rapid VFAs accumulation. Moreover, as biogas production increased, VFAs concentrations decreased rapidly. These observations indicated that although substrate degradation proceeded, the VFAs produced were rapidly consumed and did not accumulate in the system. During this stage, although total biogas production gradually declined with substrate depletion, the methane concentration remained stable at approximately 60%. Taken together, these time-series patterns suggest that by F_6 the microbial community in the GET system had shifted toward a structure favorable for efficient biomethane production.

A key point was that, after the second rice straw addition, biogas production increased sharply over a short period while VFAs were no longer detectable. Given the sampling resolution and the detection limits of the analytical method, VFAs in the GET system were likely produced continuously but turned over rapidly, resulting in pool sizes below the measurable range. Thus, after the 2nd addition of rice straw, the GET system may have approached a transient steady state in which upstream hydrolysis and acidogenesis were efficiently coupled to downstream methanogenic utilization. Under such conditions, intermediates were not expected to accumulate to detectable concentrations even when metabolic flux through the system was high.

Microbial community analyses showed that the major compositional difference between the periods surrounding the 1st and 2nd addition of rice straw was the enrichment of *Bacillus* (Figure 4). Although *Clostridium* is widely recognized as an important contributor to anaerobic cellulose degradation in many digestion systems, changes in *Clostridium* abundance in the GET system were not aligned with the observed patterns of VFAs dynamics or methane production. Likewise, archaeal abundance did not increase in parallel with methane output; notably, archaeal abundance reached its lowest level at F_8, when methane production was highest. In contrast, changes in *Bacillus* abundance were more consistently associated with methane production dynamics.

Soil bacteria have developed various strategies to adapt to changes in the soil environment. After rain or watering, soil aggregates with a radius greater than 3 mm become anaerobic environments with no oxygen (Nakano and Zuber, 1998). Under such anaerobic conditions, oxygen is replaced by different electron acceptors such as nitrate, with CO_2_ being one of them, to produce their energy.

The *Bacillus*, which was long held to be an aerobe, can also utilize a variety of electron acceptors in place of oxygen in anaerobic environments (Nakano and Zuber, 1998). Moreover, concerning the role of *Bacillus* strains in anaerobic fermentation (Islam, 2008; Sheng et al., 2010), they can play a significant role in the biodegradation of a various biomasses by producing a wide range of enzymes, such as proteases, lipases, and cellulases, which have been applied in various industrial processes (Barros et al., 2013). Subsequently, *Bacillus* produces ethanol, butanol, and other biofuels through the fermentation of various substrates (Maleki et al., 2021). Furthermore, in anaerobic fermentation, *Bacillus subtilis* is well known to produce acetic acid from acetyl-CoA via a two-step reaction, with ATP being simultaneously biosynthesized in the second step (Härtig and Jahn, 2012). According to whole genome analysis of *B. fumarioli* (Hosoyama et al., 2025), *B. fumarioli* has the two key enzymes, *i.e*., phosphate acetyltransferase (Accession: WP_066367511) and acetate kinase (Accession: WP_066370742), in relation to anaerobic fermentation from acetyl-CoA to acetic acid, indicating that *B. fumarioli* plays the same role in acetic acid production from rice straw as well as *B. subtilis* (Härtig and Jahn, 2012) and Clostridia species (Servinsky et al., 2014; Wu et al., 2015).

In *B. fumarioli*, acetic acid may be secreted outside the cell because its consumption helps maintain intracellular pH and control microbial communities (Härtig and Jahn, 2012). In an anaerobic microbial consortium under the GET system, *Methanosaeta* may use those acetic acid as a substrate for methane fermentation. The copy numbers of *B. fumarioli* and *Methanosaeta* were 10^13^ and 10^16^, respectively, suggesting that the abundance of *Methanosaeta* was 1,000 times higher (Figures 4A,E). Therefore, the reason why VFAs were not detected is due to the difference in acetic acid production by *B. fumarioli* and acetic acid consumption by *Methanosaeta*, which is caused by the difference in copy numbers between *B. fumarioli* and *Methanosaeta*.

Taken together, the obtained results of this study suggested that the high biogas production (300 L/kg of rice straw) of the GET system was as follows:

(1)    VFAs such as acetic acid, which are necessary for biogas production, were not detected.
(2)    Analysis of microbial community structure revealed that Bacillus bacteria were predominant over Clostridium bacteria.
(3)    Among archaea, *Methanosaeta* was predominant.
(4)    Quantitative real-time PCR results showed that the increase in biogas production was synchronized with the copy number of *B. fumarioli*.
(5)    *Methanosaeta* had the highest copy number, 1,000 times that of *B. fumarioli*. Furthermore, based on existing knowledge, the following was clear about the *B. fumarioli* identified in this study:
(6)    Bacillus bacteria can biosynthesize acetic acid under anaerobic environments (Härtig and Jahn, 2012).
(7)    Whole genome analysis of *B. fumarioli* revealed that it possesses a gene cluster capable of acetic acid biosynthesis (Hosoyama et al., 2025).

**FIGURE 5:**
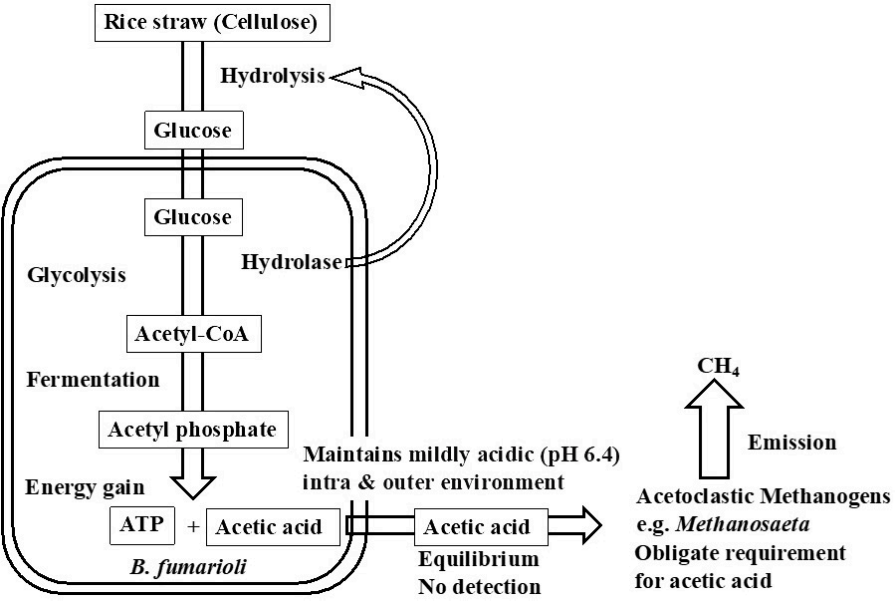
The schematic diagram of the GET system operation on methanogenesis.

Based on the comprehensive and collective knowledge, the characteristics of the high biogas production of the GET system were suggested as summarized in Figure 5.

The GET system is an innovative technology that not only reduces methane emissions from paddy fields but also contributes to renewable energy production with only rubber sheets and gas collection bags. The abundance of *B. fumarioli* became the key biomarker to confirm whether the methane fermentation process is working properly in the GET system. Therefore, further research on *B. fumarioli* will pave important avenues not only for fundamental research into the regulatory mechanisms of anaerobic metabolism but also for applied research, such as promoting renewable energy and discovering new enzymes for recycling cellulosic wastes.

## Data Availability

The datasets presented in this study can be found in online repositories. The names of the repository/repositories and accession number(s) can be found in the article/Supplementary material.
