# Supplementary material for: Dynamics and function of the GET system microbial community: insights into the role of the genus Bacillus in biogas production
Source: Front Microbiol. 2026 Feb 9;17:1729398. doi: 10.3389/fmicb.2026.1729398 (PMC12926373; doi:10.3389/fmicb.2026.1729398)
Supplement: Supplementary file 1 [file Table_1.docx]

**Supplement Tables and Figures**

**Table S1 HPLC analysis conditions.**

| **System controller** | SCL-10Avp |  | |  | |
| --- | --- | --- | --- | --- | --- |
| **Pump A, B** | LC-ADvp | **Pump A flow rate**  **Pump B flow rate**  **Pressure limit upper limit**  **Pressure limit lower limit** | | 0.8 ml/min  0.8 ml/min  80 kgf/cm^2^  0 kgf/cm^2^ | |
| **Autosampler** | SIL-HTC | **Needle stroke**  **Sample injection amount**  **Sample suction speed**  **Sample storage temperature** | | 52 mm  50 μl  15 μl/sec  4℃ | |
| **Mobile phase liquid** | 5 mM  *p*-toluenesulfonic acid | | | | |
| **Buffer solution** | 5 mM *p*-toluenesulfonic acid  20 mM Bis-Tris 100 μM EDTA | | | | |
| **Column oven** | CTO-10Acvp | **Temperature** | | 40 °C | |
| **Analytical column** | Shim-pack  SCR-102H  (L: 300 mm × I D: 8.0 mm Double) | | | | |
| **Guard column** | Shim-pack  SCR-102H (G)  (L: 50 mm ×ID: 7.8 mm) | | | | |
| **Detector** | CDD-10Avp  Electrical  conductor detector | | **Analysis time** | | 55 min |
| **Data processing equipment** | LC-solution | | | | |
| **Quantitative method** | External standard method  (Conc. 200 ppm) | | | | |

**Table S2 Primer used for qPCR.**

| Primer | Sequence (5’-3’) |
| --- | --- |
| *B.fumarioli f* ^*1^ | CGGGTCGTAAAGCTCTGTTG |
| *B.fumarioli r*^*2^ | CCGTGGCTTTCTGGTTAGGT T |
| *B.flexus f* | CAGCCCCACCCTTGACTTT |
| *B.flexus r* | CGGCCATTGTATGACGTGTG |
| *C.butyricum f* | ATGCAGCACCCAAGTTAAGC |
| *C.butyricum r* | AGCGTTGTCCGGATTTACTG |
| *C. bowmani f* | TAAATCCGGACAACGCTTGC |
| *C. bowmani r* | GACGGTCTTCGGATTGTAAAGC |
| *B.cereus_left*  *B.cereus_right* | *CAGCCCCACCCTTGACTTT*  *CGGCCATTGTATGACGTGTG* |
| McrA159F  McrA345R | AAAGTGCGGAGCAGCAATCACC  TCGTCCCATTCCTGCTGCATTGC |

^*^1: forward; ^*^2: reverse

**
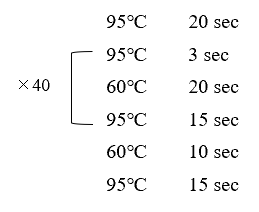
Table S3 Reaction composition of real-time PCR.**

| Fast SYBR Green Master Mix | 10.0 µL |
| --- | --- |
| Template (10 ng/1.6 µL) | 1.6 µL |
| F primer (10 µM) | 0.4 µL |
| R primer (10 µM) | 0.4 µL |
| RT-PCR Grade Water | 7.6 µL |
| Total | 1. L |

**
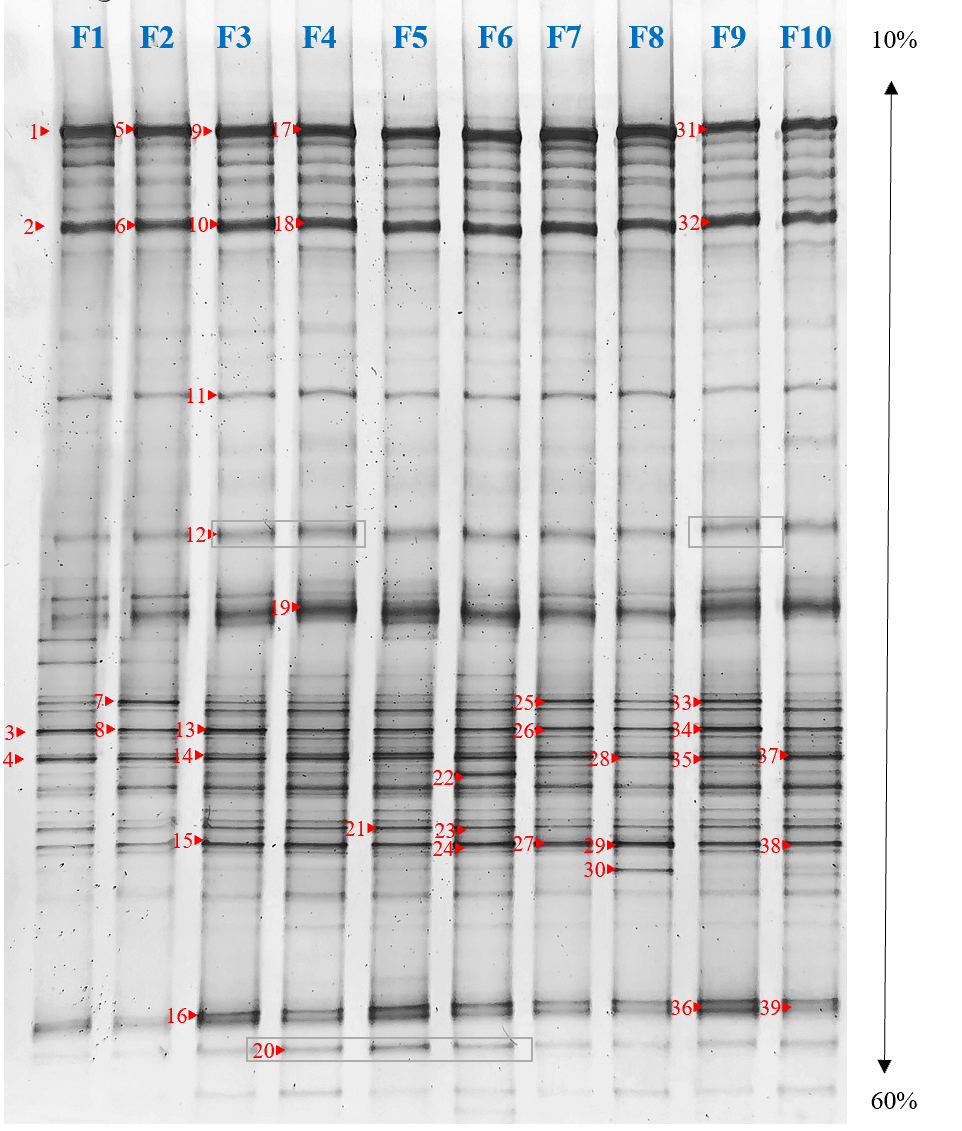
**

**Figure S1 Dynamics analysis of the genus *Clostridium* community by DGGE.**

**Table S4 *Clostridium* with more than 97% homology**

| Band | Related species | Assession No. | Similarity (%) |
| --- | --- | --- | --- |
| 2 | *Clostridium puniceum* strain BL 70/20 | NR_026105.1 | 97.55 |
| 3 | *Clostridium puniceum* strain BL 70/20 | NR_026105.1 | 99.38 |
| 4 | *Clostridium puniceum* strain BL 70/20 | NR_026105.1 | 97.96 |
| 6 | *Clostridium saccharobutylicum* strain NCP 262 | NR_121710.1 | 97.52 |
| 7 | *Clostridium saccharobutylicum* strain NCP 262 | NR_122051.1 | 99.38 |
| 8 | *Clostridium puniceum* strain BL 70/20 | NR_026105.1 | 98.76 |
| 10 | *Clostridium butyricum* strain JCM 1391 | NR_113244.1 | 97.29 |
| 13 | *Clostridium chromiireducens* strain GCAF-1 | NR_122090.1 | 98.76 |
| 15 | *Clostridium bowmanii* strain A-1/C-an/C1 | NR_037091.1 | 98.19 |
| 18 | *Sarcina ventriculi* strain DSM 286 | NR_026146.1 | 97.06 |
| 19 | *Clostridium magnum* DSM 2767 | NR_119084.1 | 97.89 |
| 24 | *Clostridium psychrophilum* strain A-1/C-an/I | NR_037090.1 | 97.74 |
| 25 | *Clostridium saccharobutylicum* strain P 262 | NR_03695.1 | 98.18 |
| 26 | *Clostridium chromiireducens* strain GCAF-1 | NR_122090.1 | 98.32 |
| 27 | *Clostridium frigoris* strain D-1/D-an/II | NR_036822.1 | 97.24 |
| 29 | *Clostridium psychrophilum* strain A-1/C-an/I | NR_037090.1 | 97.94 |
| 30 | *Sarcina ventriculi* strain DSM 286 | NR_026146.1 | 97.29 |
| 32 | *Clostridium saccharobutylicum* strain NCP 262 | NR_122051.1 | 98.37 |
| 33 | *Clostridium puniceum* strain BL 70/20 | NR_026105.1 | 97.98 |
| 35 | *Clostridium psychrophilum* strain A-1/C-an/I | NR_037090.1 | 97.56 |
| 38 | *Clostridium bowmanii* strain A-1/C-an/C1 | NR_037091.1 | 98.00 |

**
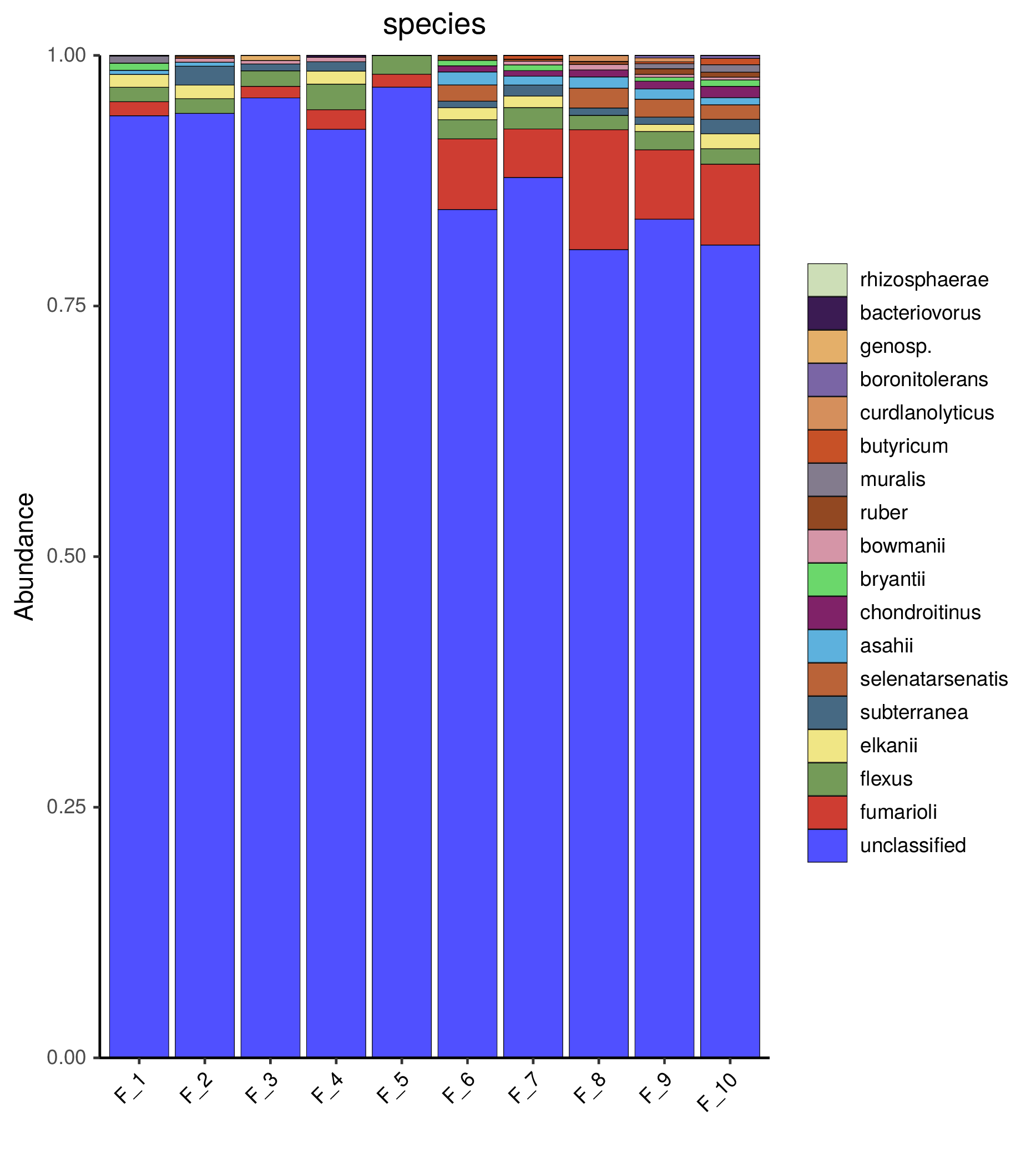
 Figure S2 Species-level clustering tree and histogram combination analysis chart**
